# Immune Priming and Long-term Persistence of Memory B Cells After Inactivated Poliovirus Vaccine in Macaque Models: Support for at least 2 Doses

**DOI:** 10.1093/cid/ciy634

**Published:** 2018-10-30

**Authors:** Siddhartha Kumar Bhaumik, Raveendra R Kulkarni, William C Weldon, Eduardo L V Silveira, Hasan Ahmed, Sivaram Gunisetty, Anmol Chandele, Rustom Antia, Harish Verma, Roland Sutter, Mark A Pallansch, M Steven Oberste, Francois Villinger, Walter Orenstein, Kaja Murali-Krishna

**Affiliations:** 1 Department of Pediatrics, Division of Infectious Diseases, Emory University School of Medicine, Atlanta, Georgia; 2 Division of Viral Diseases, Centers for Disease Control and Prevention (CDC), Atlanta, Georgia; 3 Yerkes Primate Center, Emory University School of Medicine, Atlanta, Georgia; 4 Department of Biology, Emory University, Atlanta, Georgia; 5 ICGEB-Emory Vaccine Center, International Center for Genetic Engineering and Biotechnology, Aruna Asaf Ali Marg, New Delhi, India; 6 Emory Vaccine Center, Emory University School of Medicine, Atlanta, Georgia; 7 Polio Eradication Department, World Health Organization, Geneva, Switzerland; 8 Department of Medicine, Emory University School of Medicine, Atlanta, Georgia

**Keywords:** polio, IPV, memory, priming, B-cells, neutralizing antibodies

## Abstract

**Background:**

As a risk-mitigation strategy to minimize paralytic polio following withdrawal of Sabin type 2 from the oral poliovirus vaccine in April 2016, a single full dose or 2 fractional doses of inactivated poliovirus vaccine (IPV) are recommended. However, limited knowledge exists on long-term persistence of immune memory following 1- or 2-dose IPV schedules.

**Methods:**

We examined induction and maintenance of immune memory following single- vs 2-dose IPV schedules, either full-dose intramuscular or fractional-dose intradermal, in rhesus macaques. Humoral responses, bone marrow–homing antibody-secreting plasma cells, and blood-circulating/lymph node–homing memory B cells were examined longitudinally.

**Results:**

A single dose of IPV, either full or fractional, induced binding antibodies and memory B cells in all vaccinated macaques, despite failing to induce neutralizing antibodies (NT Abs) in many of them. However, these memory B cells declined rapidly, reaching below detection in the systemic circulation by 5 months; although a low frequency of memory B cells was detectable in draining lymph nodes of some, but not all, animals. By contrast, a 2-dose vaccination schedule, either full or fractional, efficiently induced NT Abs in all animals along with bone marrow–homing plasma cells and memory B cells. These memory B cells persisted in the systemic circulation for up to 16 months, the maximum duration tested after the second dose of vaccination.

**Conclusions:**

Two doses of IPV, regardless of whether fractional or full, are more effective than a single dose for inducing long-lasting memory B cells.

With wild polioviruses (WPV) nearing eradication worldwide, it is important to develop and implement polio endgame strategies to sustain eradication [1, 2]. Oral polio vaccine (OPV) that consists of live attenuated poliovirus types 1, 2, and 3 (Sabin 1, 2, and 3) historically served as the most efficient, cost-effective, and easy-to-implement vaccination strategy. However, in extremely rare instances, the OPV can cause vaccine-associated paralytic polio (VAPP) in vaccinated individuals or their close contacts. Additionally, continued use of OPV in the setting of low population immunity can result in generation of vaccine-derived polio viruses (VDPV) that, through circulation, acquire the transmissibility and neurovirulence properties of WPV (circulating VDPV [cVDPV]), leading to outbreaks of polio [3]. Alternatively, immune-deficient vaccine-derived polioviruses (iVDPVs) may originate and be chronically shed from immunodeficient individuals exposed to the Sabin vaccine. While type 2 WPV has been declared eradicated, the most common cVDPV outbreaks were caused by serotype 2, which can sometimes lead to paralysis in vaccine recipients or close contacts. As a risk mitigation strategy against the emergence of such VDPVs, the World Health Organization Strategic Advisory Group of Experts on Immunization (SAGE) has recommended that OPV usage must be phased out. Accordingly, during the last 2 weeks of April 2016, there was a global switch from trivalent OPV (tOPV) containing all 3 types to bivalent OPV (bOPV) containing only types 1 and 3 [4]. Simultaneously, introduction of at least 1 dose of inactivated poliovirus vaccine (IPV) has also been recommended to elicit protection against the emergence and/or continued circulation of cVDPVs, as well as potential reintroductions of WPVs (eg, a break in laboratory containment) [5]. Although inefficient when compared to OPV in inducing intestinal mucosal immunity [6–8], IPV is capable of inducing excellent systemic immunity, conferring protection against paralytic disease without the associated risks of VAPP or cVDPV emergence.

Many developed countries have already shifted from OPV to exclusive use of IPV in the past few decades. Wherever this complete shift has occurred, IPV has been implemented in a 3- to 7-dose vaccination schedule. For example, the routine IPV vaccination schedule in the United States calls for doses at the ages of 2 months, 4 months, 6–18 months, and 4–6 years. Such elaborate schedules may be impractical in many developing countries for reasons such as limited IPV supplies, high IPV cost compared to OPV, and implementation challenges due to the requirement for skilled personnel to administer a vaccine that requires injections [9]. Considering these issues, SAGE has recommended at least 1 IPV dose to be implemented in all countries as part of the effort that led to the phasing out of trivalent OPV. Because of current IPV shortages, a 2-dose schedule consisting of a one-fifth dose of IPV via intradermal delivery has also been considered. This recommendation is supported by studies showing a 2 fractional dose schedule delivered via the intradermal route was equivalent to or superior to 1 intramuscular full dose in inducing systemic immunity [10–12]. Although it is well recognized that a single IPV dose is insufficient to induce neutralizing antibodies (NT Abs) in a substantial proportion of vaccinees, it can actually prime the immune system, perhaps by inducing memory B cells that can contribute to an anamnestic response upon reexposure to poliovirus antigens. Thus, it is suggested that single-dose IPV-primed persons who have not seroconverted might have some protection against paralytic poliomyelitis [13–19].

Nevertheless, uncertainties remain, such as whether the memory B cells generated by a single dose of IPV persist long term, whether the memory B cells induced by full-dose i.m. vs fractional dose i.d. vaccine delivery behave similarly in their long-term persistence characteristics, whether the long-term persistence of the memory B cells was equally robust against each of the 3 poliovirus serotypes, and whether a 2-dose IPV schedule is superior compared to a single dose of IPV in eliciting long-lasting memory cells. These issues are difficult to address directly in humans since the countries that have shifted to 1- or 2-dose IPV schedules continue to use bOPV containing types 1 and 3. Consequently, the ongoing transmission of these OPV-related viruses to IPV vaccinees can confound the ability to evaluate the immunity induced by IPV alone. In this study, we used a macaque model to gain a detailed understanding of these issues. In addition, the macaque model allowed evaluation of bone marrow–homing plasma cells and lymphoid tissue–homing memory B cells, which is a difficult, if not impossible, task to be carried out in humans.

## METHODS

### Experimental Design

**Figure 1.**
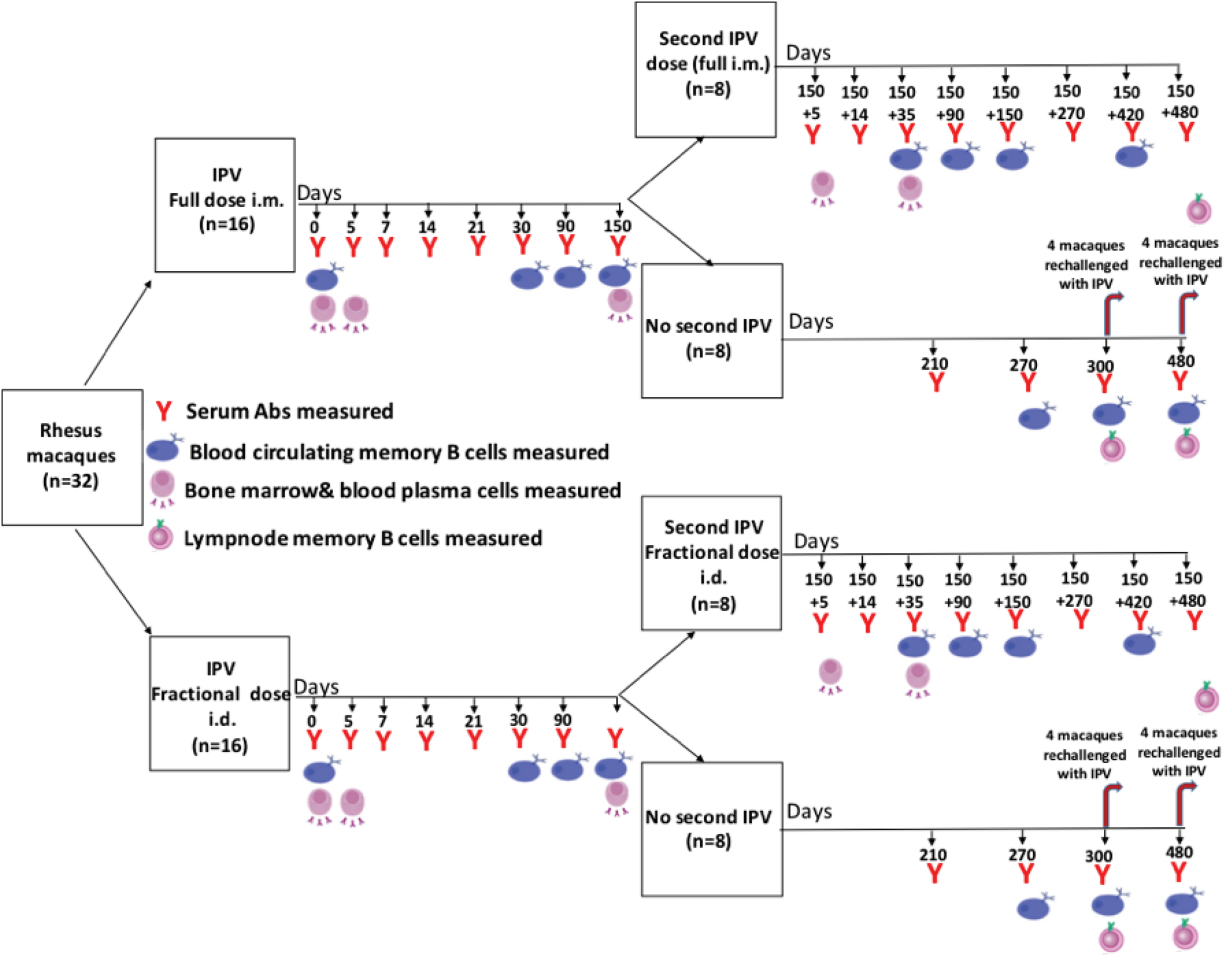
Overall experimental design. Abbreviations: Abs, antibodies; i.d., intradermal; i.m., intramuscular; IPV, inactivated poliovirus vaccine.

A schematic of the overall experimental design is shown in Figure 1. A total of 32 rhesus macaques, aged 2–8 years, were included in the study. Of these 32 macaques, 16 were immunized with IPV full-dose intramuscular and 16 were immunized with a one-fifth fractional dose of IPV intradermal. In each of the 16 full-dose and 16 fractional-dose macaques, half (8) received a single dose of IPV and the other half (8) received 2 doses of IPV at a 5-month interval. In the single-dose group, 4 macaques were challenged with IPV on day 300 and 4 were challenged with IPV on day 480 to assess whether they retained the immune priming effect long term after a single dose of IPV. Humoral responses, blood-circulating and lymph node–homing memory B cells, and bone marrow–homing/blood-circulating plasma cells specific to each of the 3 poliovirus serotypes were analyzed longitudinally at the indicated time points.

### Inactivated Polio Vaccine

IPV in vaccine vials was purchased from Sanofi Pasteur (IPOL). The dose was the same as used in humans, corresponding to 0.5 mL and composed of 40 D antigen units of type 1, 8 D antigen units of type 2, and 32 D antigen units of type 3 poliovirus. The fractional IPV dose corresponded to 0.1 mL, composed of 8 D antigen units of type 1, 1.6 D antigen units of type 2, and 6.4 D antigen units of type 3 poliovirus. Immunizations and sample collections were performed in a staggered manner so that a maximum of 4 macaques were handled on a given day. Blood was collected at serial time points, whereas bone marrow aspirates and draining lymph nodes were collected at select time points as indicated in Figure 1.

#### Serological Assays

Anti-poliovirus NT Abs against each of the 3 poliovirus serotypes were measured using the modified poliovirus microneutralization assay as previously described [20]. Anti-poliovirus antigen-binding antibodies were detected by overnight coating of Nunc Maxisorp enzyme-linked immunosorbent assay plates with each of the monovalent inactivated IPV component antigens (Sanofi Pasteur) at a concentration of 1 µg/mL in phosphate-buffered saline (PBS) followed by incubation with 3-fold serially diluted heat-inactivated plasma. The polio antigen-specific antibodies were detected using peroxidase-conjugated anti-monkey immunoglobulin (Ig) M or IgG antibodies (Rockland) and developed using 3,3′,5,5′-tetramethylbenzidine (TMB) substrate (Biorad).

### Lymphocyte Isolation

The peripheral blood mononuclear cells and plasma were isolated by centrifuging the peripheral blood collected in Becton Dickinson Cell Preparation Tube (CPT) tubes at 1600 × *g* for 30 min with no acceleration and deceleration at room temperature [21]. For the bone marrow lymphocyte preparations, the bone marrow aspirates were first passed through a cell strainer followed by an underlayer of histopaque-1077 and centrifuged at 800 × *g* for 30 minutes with no acceleration and deceleration at room temperature. The interface was then collected, red blood cells (RBCs) were lysed, and the remaining cells were washed and resuspended in 1640 Roswell Park Memorial Institute 1640 medium (RPMI) containing 10% fetal bovine serum, penicillin/streptomycin, and L-glutamine. For the lymph node lymphocyte isolation, small pieces of lymph node biopsies were mashed on a cell strainer with a syringe plunger. RBCs were lysed, and the suspension was washed and resuspended in complete RPMI as described for the memory B cell assay.

### ELISPOT Assays

Antibody-secreting cells (ASCs) in blood and bone marrow were analyzed by performing the enzyme-linked immunospot assay (ELISPOT) as described in detail elsewhere [21]. Briefly, 96-well MultiScreen_HTS_ HA filter plates (Millipore) were coated with 10 µg/mL of anti-monkey IgG (H&L) antibody (Rockland) to determine the total ASCs or with 10 µg/mL of polio virus–specific antigens (Sanofi Pasteur) to determine the antigen-specific ASCs. After the overnight coat at 4°C, the plates were washed with PBS/0.05% Tween 20 (PBS-T) followed by PBS and blocked for 2 hours with complete RPMI at 37°C. The lymphocytes were then plated with 3-fold serial dilutions and kept in a 5% CO_2_ incubator at 37°C for 5 hours. The plates were then washed with PBS followed by PBS-T and incubated with biotin-conjugated anti-monkey IgG (Rockland) for 2 hours at room temperature. The plates were washed with PBS-T and incubated for 3 hours at room temperature with Horseradish Peroxidase Avidin D (Avidin D-HRP; Vector labs). The plates were then washed with PBS-T followed by PBS and developed with 3-amino-9-ethylcarbazole (AEC) substrate (BD Biosciences) according to the manufacturer’s protocol. Plates were then dried, and spots were imaged and counted using Immunospot Cellular Technology Limited (CTL) counter and Image Acquisition 4.5 software (Cellular Technology).

### Memory B Cell Assays

The lymphocytes were stimulated in 48-well plates with 1 × 10^6^ cells in 1 mL of mitogen stimulation medium per well. One milliliter of mitogen stimulation medium constitutes 0.1 µL of *Staphylococcus aureus* Cowan (Sigma), 6 µL of CpG-2006 (Invivogen), 1 µL of beta-mercaptoethanol, 1 µL of pokeweed mitogen (MP Biomedical), and complete RPMI medium. Following 5 days of stimulation in 5% CO_2_ at 37°C, the ELISPOT assay was performed as described above to determine the frequency of antigen-specific memory B cells.

### Statistics

Data are reported for individual macaques at specific time points mentioned along with their geometric mean and standard deviation. All graphs were generated using Prism 6.0 (GraphPad Software).

## RESULTS

**Figure 2.**
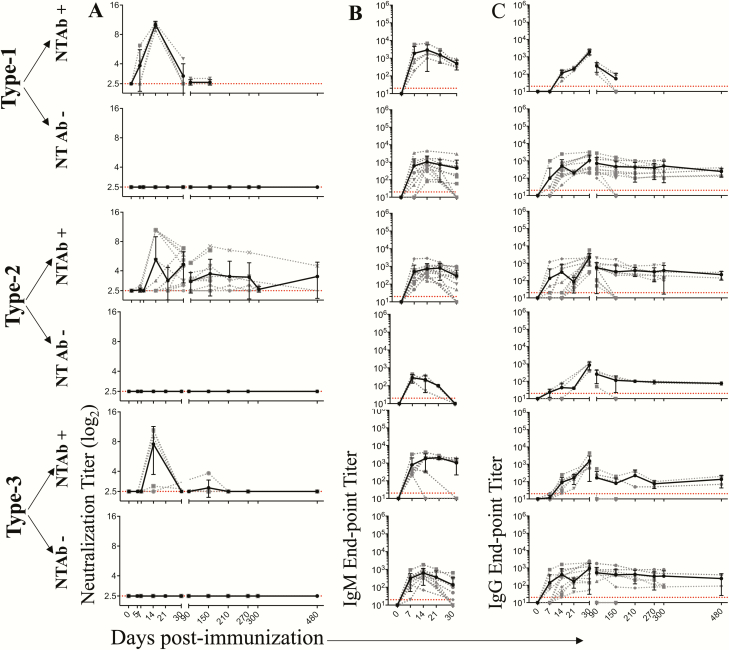
Longitudinal analysis of humoral immunity following a single intramuscular full-dose inactivated poliovirus vaccine (IPV) immunization. Neutralization titers *(A*), immunoglobulin (Ig) M antibody (Ab) binding titers *(B*), and IgG Ab binding titers *(C*) specific to each of the poliovirus serotypes were measured. The macaques were divided into 2 categories: neutralizing (NT) (Ab) inducing (NT Ab+) and NT Ab noninducing (NT Ab−) based on detectable NT Ab titers within the first 30 days post single IPV dose immunization. The dashed lines indicate data for individual macaques, and the solid line indicates the geometric mean of the titers of individual macaques at the particular time post-vaccination. Of the 16 macaques immunized with single intramuscular full-dose IPV, all 16 were analyzed at days 0, 5, 7, 14, 21, 30, 90, and 150; 8 were analyzed at days 210, 270, and 300; and 4 were analyzed at day 480. Dotted red lines indicate limit of detection. Abbreviations: Ig, immunoglobulin; NT Abs, neutralizing antibody.

**Figure 3.**
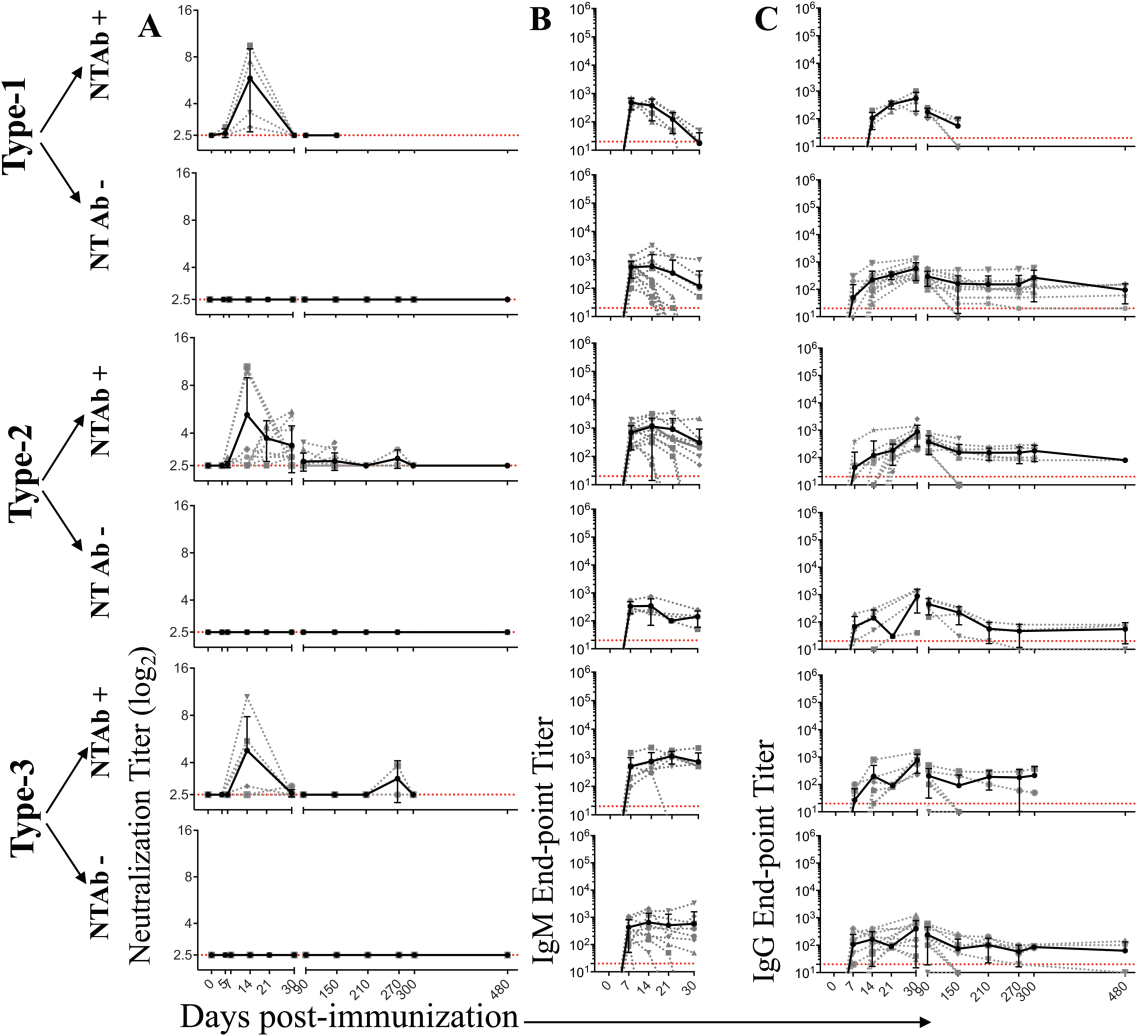
Longitudinal analysis of humoral immunity following a single intradermal fractional-dose inactivated poliovirus vaccine (IPV) immunization. Neutralization titers *(A*), immunoglobulin (Ig) M antibody (Ab) binding titers *(B*), and *(C*) IgG Ab binding titers *(C*) specific to each of the poliovirus serotypes were measured. The macaques were divided into 2 categories: neutralizing (NT) Ab) inducing (NT Ab+) and NT Ab noninducing (NT Ab−) based on detectable NT Ab titers within the first 30 days post single IPV dose immunization. The dashed lines indicate data for individual macaques, and the solid line indicates the geometric mean of the titers of individual macaques at the particular time post-vaccination. Of the 16 macaques immunized with single intradermal fractional-dose IPV, all 16 were analyzed at days 0, 5, 7, 14, 21, 30, 90, and 150; 8 were analyzed at days 210, 270, and 300; and 4 were analyzed at day 480. Dotted red lines indicate limit of detection. Abbreviations: Ig, immunoglobulin; NT Abs, neutralizing antibody.

Similar to what has been reported in human studies, a single IPV dose induced detectable NT Abs in only a fraction of the immunized macaques (Table 1). The fraction of animals in which detectable NT Abs was induced was highest for type 2, followed by type 3 and type 1. This hierarchy was similar in macaques that received a single full dose of IPV (i.m.) or a single fractional dose of IPV (i.d.). In order to understand long-term persistence of the NT Abs (in situations where they were induced), we performed a longitudinal analysis for up to 16 months post-immunization in macaques that received a single full dose of IPV (i.m.) (Figure 2) and in macaques that received a single dose of fractional IPV (i.d.) (Figure 3). NT Ab titers were plotted for individual macaques (dotted lines) or the geometric mean of the group (solid lines). For analysis of each serotype, we subdivided the macaques into 2 groups: a group in which NT Abs were induced (NT Ab+) and a group in which NT Abs were not induced (NT Ab−). Where induced, the NT Ab titers peaked around day 14 but quickly declined and fell below detection levels by day 30 for type 1 and by day 210 for type 3. For type 2, the titers dropped substantially but persisted longer. These patterns were strikingly similar in single-dose full IPV (Figure 2A) vs single-dose fractional IPV (Figure 3A). Taken together, these results suggest that with a single dose of IPV, irrespective of full or fractional dose, NT Abs are not induced in a majority of the animals to types 1 and 3 and, when induced, they fail to persist long term for poliovirus types 1 and 3 or the titers drop substantially over time for poliovirus type 2.

**Table 1.** Induction of Neutralizing Antibodies Following a Single Intramuscular Full-dose or Single Intradermal One-fifth Dose Inactivated Poliovirus

Polio Virus Subtype Specificity	Number of Macaques per Group and Percent of the Macaques Inducing Detectable NT Abs Following a Single Full Dose (Intramuscular) IPV^a^	Number of Macaques per Group and Percent of the Macaques Inducing Detectable NT Abs Following a Single Fractional Dose (Intradermal) IPV^a^
Sabin-1	4/16 (25%)	4/16 (25%)
Sabin-2	13/16 (81.2%)	12/16 (75%)
Sabin-3	6/16 (37.5%)	6/16 (37.5%)

Abbreviations: IPV, inactivated poliovirus vaccine; NT Abs, neutralizing antibodies.

^a^Detectable NT Abs are defined as serum titers above log_2_ between 7 and 30 days post IPV immunization.

To determine whether the lack of induction of detectable NT Abs necessarily means lack of induction of antibodies, we examined the production of binding IgM (Figures 2B and 3B) and IgG (Figures 2C and 3C) antibodies specific to each of the 3 poliovirus-derived antigens. Both IgM and IgG antibodies were induced in all the vaccinees irrespective of whether NT Abs were induced or not. The pattern was similar in both full-dose and fractional-dose vaccine recipients. These results suggest that a single dose of IPV induced humoral response in all the animals irrespective of whether it was capable of inducing NT Abs or not.

**Figure 4.**
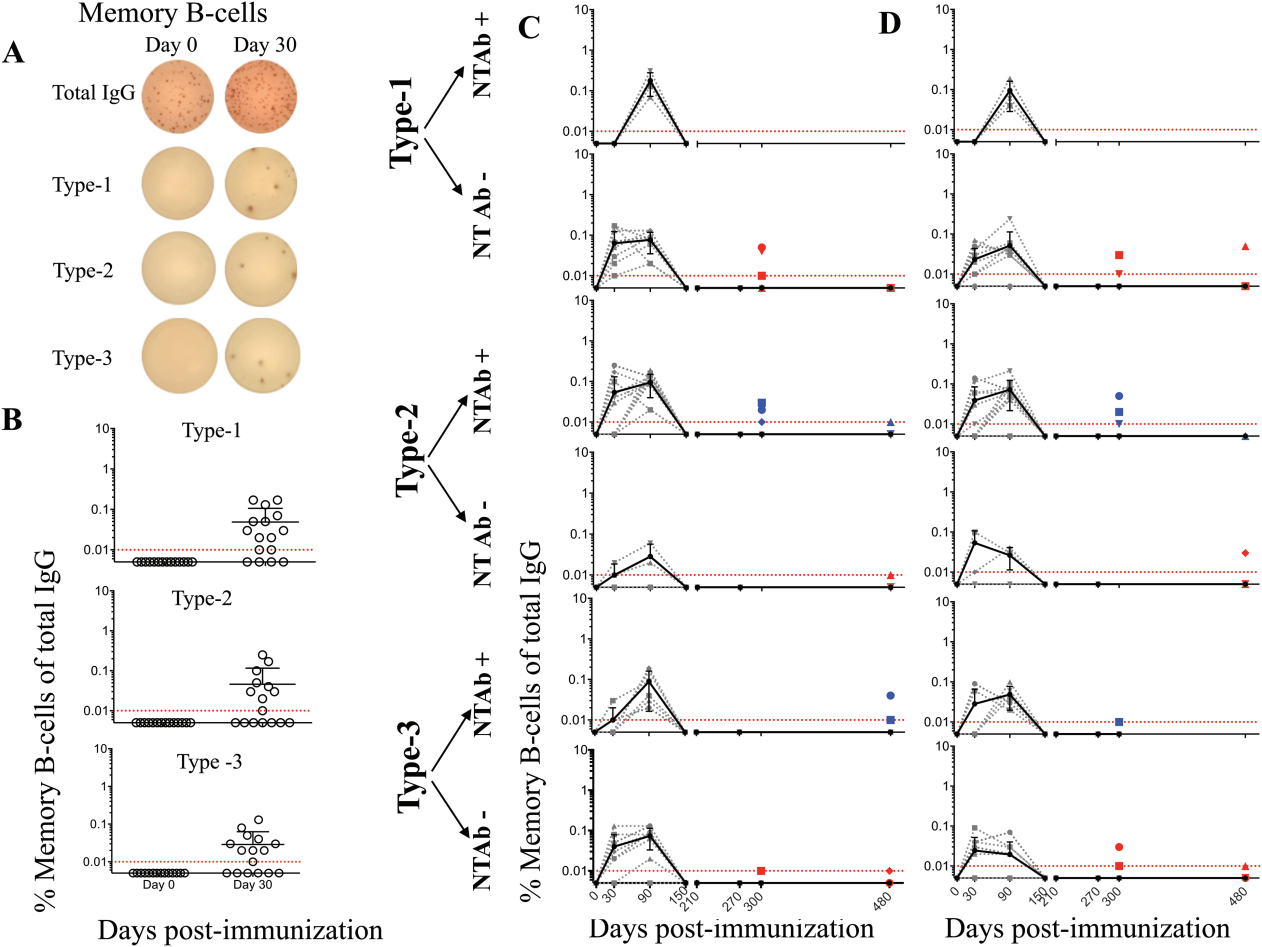
Memory B cell (MBC) induction and persistence following a single inactivated poliovirus vaccine (IPV) immunization. Example of MBC enzyme-linked immunospot assay *(A*), frequencies of MBCs specific to each of the poliovirus serotypes in unimmunized vs day 30 post single full-dose IPV intramuscular (i.m.) immunized macaques *(B*), longitudinal analysis of blood-circulating MBCs in single full-dose IPV i.m. immunized macaques *(C*), and longitudinal analysis of blood-circulating MBCs in single fractional-dose IPV intradermal immunized macaques *(D*) are shown. In panels *C* and *D*, macaques were divided into 2 categories: neutralizing antibody (NT Ab) inducing (NT Ab+) or NT Ab noninducing (NT Ab−) based on detectable NT Ab titers within the first 30 days post single IPV dose immunization. The dashed lines indicate data for individual macaques, and the solid line indicates the geometric mean of the MBC frequencies of individual macaques at the particular time post-vaccination. Of the 16 macaques immunized in each of the full or fractional groups, all 16 were analyzed at days 0, 30, 90, and 150; 8 were analyzed at days 210, 270, and 300; and 4 were analyzed at day 480. Dotted red lines indicate limit of detection. The colored dots show lymph node MBC frequencies in 4 macaques on day 300 and in 4 macaques on day 480. Blue dots indicate individual macaques that generated neutralization titers after the first dose of IPV, and the red dots indicate individual macaques that failed to generate neutralization titers after the first dose of IPV. The shape of the symbol used to indicate lymph node MBC in a given macaque corresponds to the shape of the symbol used to indicate MBC of the circulation in the same macaque. Abbreviations: Ig, immunoglobulin; NT Abs, neutralizing antibody.

To determine whether memory B cells are induced and maintained by single-dose full or fractional IPV, we characterized memory B cells specific to each of the poliovirus serotype antigens in the blood circulation (at multiple time points) and in lymph node tissue (in selected macaques at limited time points) (Figure 4). Memory B cells were detected in the blood of all animals and peaked around day 90 post-vaccination irrespective of whether or not they induced NT Abs. However, these memory B cells declined rapidly thereafter, falling below detection by day 150. Interestingly, even though memory B cells could not be detected in the systemic circulation at later times, a low frequency of memory B cells was detectable in draining lymph nodes. This low frequency seen in the lymph nodes also declined with time, although there was heterogeneity between individual animals. These patterns were similar in single-dose full IPV (i.m.) (Figure 4C) vs single-dose fractional IPV (i.d.) animals (Figure 4D). Taken together, these results suggest that a single dose of IPV, irrespective of full dose or fractional dose, induced memory B cells in all the animals. However, these memory B cells failed to persist long term in the systemic circulation.

**Figure 5.**
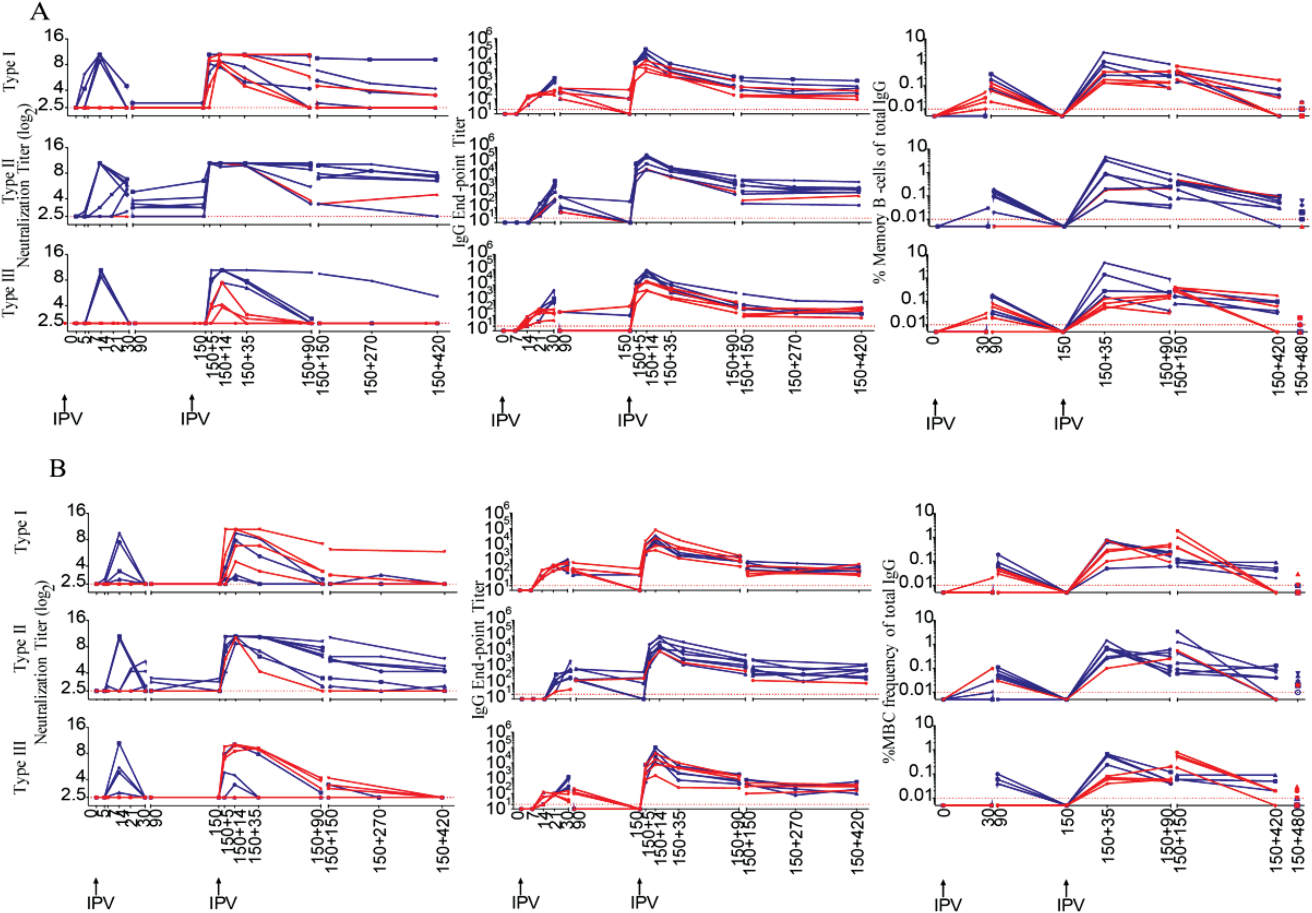
Assessment of long-term memory with 2-dose inactivated poliovirus vaccine (IPV). *(A*) Two-dose full (intramuscular) IPV. *(B*) Two-dose fractional (intradermal) IPV. Neutralizing antibody titers (left panels), immunoglobulin G titers (middle panel), and memory B cell frequencies (right panel) specific to each of the poliovirus serotypes are shown. Second dose of IPV was given 5 months post first dose. Blue lines indicate individual macaques that generated neutralization titers after the first dose of IPV, and the red lines indicate individual macaques that failed to generate neutralization titers after the first dose of IPV. Unjointed dots in the right-most panel at day 150 + 480 indicate the frequencies of draining lymph node memory B cells specific to each of the poliovirus serotypes. Abbreviations: Ig, immunoglobulin; IPV, inactivated poliovirus vaccine.

By marked contrast to the single dose of IPV, a 2-dose IPV schedule was able to induce NT Abs in all the macaques (Figure 5A and B). As expected with anamnestic responses, the time taken for NT Ab induction was much faster after the second dose, with titers also substantially higher after the second dose compared to the first dose. In a majority of the animals, NT Abs were detected as early as day 5 post second dose, compared to the 14 or more days taken for the detection of NT Abs (where induced) after a single immunization. Type 2 specific NT Abs induced by the 2-dose IPV schedule persisted for up to 420 days post second dose (the maximum duration tested in these experiments). This long-term persistence was variable for type 1 and type 3. The 2 full doses of IPV (i.m.) induced type 1 specific NT Abs, which persisted for up to 420 days post second dose in 4 macaques, while they started declining from day 150 in 2 macaques. The 2-dose fractional IPV (i.d.) induced type 1 specific NT Abs, which persisted for 420 days post second dose in 1 macaque, while they started declining from day 150 in 4 macaques. Type 3 specific NT Abs started declining from day 150 post second dose in both 2-dose full IPV (i.m.) and 2-dose fractional IPV (i.d.) animals. Similar to NT Abs, the IgG binding antibodies were also induced much more rapidly post second dose. The frequency of memory B cells induced after the second dose was approximately 1 log higher compared to the frequency of memory B cells induced after the first dose. More importantly, the memory B cells induced by the 2-dose IPV schedule were long-lasting (for up to 420 days post second dose, the maximum duration tested in these experiments) in the systemic circulation of a majority of the macaques, unlike the pattern seen following a single dose of IPV. Taken together, these results demonstrate that a single dose of IPV is capable of inducing memory B cells in all the animals, but these memory B cells fail to persist long-term in the systemic circulation. Two doses of IPV induce much higher levels of memory B cells compared to a single dose; these 2-dose induced memory B cells persist longer term. When used in a 2-dose schedule, both full-dose i.m. or fractional-dose i.d. are equally efficient in the ability to induce these longer-term persisting memory B cells.

**Figure 6.**
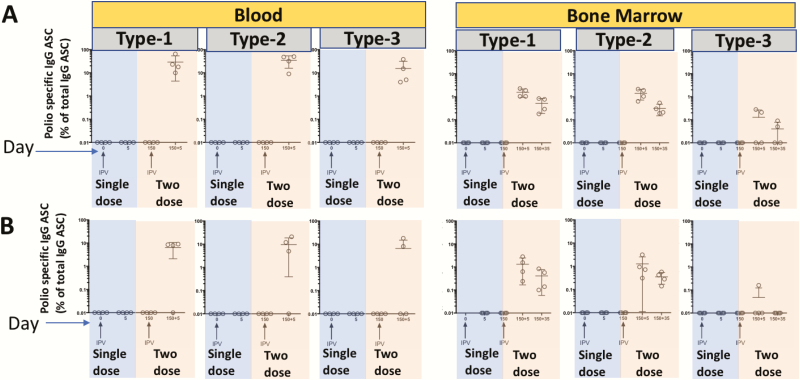
Enumeration of antibody-secreting plasma cells (ASCs) in single vs 2-dose inactivated poliovirus vaccine (IPV) immunization. *(A*) Full-dose IPV intramuscular immunized macaques. *(B*) Fractional-dose IPV intradermal immunized macaques. ASCs were enumerated as percent of total immunoglobulin G–secreting cells in blood or bone marrow against poliovirus type 1, type 2, and type 3 specificities. Abbreviations: ASC, antibody-secreting plasma cell; Ig, immunoglobulin; IPV, inactivated poliovirus vaccine.

Bone marrow seeding of the isotype-switched antibody-secreting cells (ASC) is important for long-term maintenance of humoral memory. Consistent with the ineffective induction and maintenance of serum NT Abs, we found that polio type 1, 2, or 3 specific IgG ASCs were below detection in blood circulation or in bone marrow aspirates following either a single full dose of IPV (i.m.) or a single fractional dose of IPV (i.d.) (Figure 6). By contrast, a 2-dose IPV schedule induced a markedly antigen-specific ASC response in both blood and bone marrow at day 5 post second dose, and this response persisted in bone marrow even at 35 days post second dose (ie, day 150 + 35, the maximum time tested for this analysis in these experiments). Strikingly similar patterns were observed with full- vs fractional-dose IPV. Thus, a single dose of IPV was highly inefficient in inducing the bone marrow seeding ASC compared to 2 doses of IPV in both full-dose and fractional-dose schedules.

**Figure 7.**
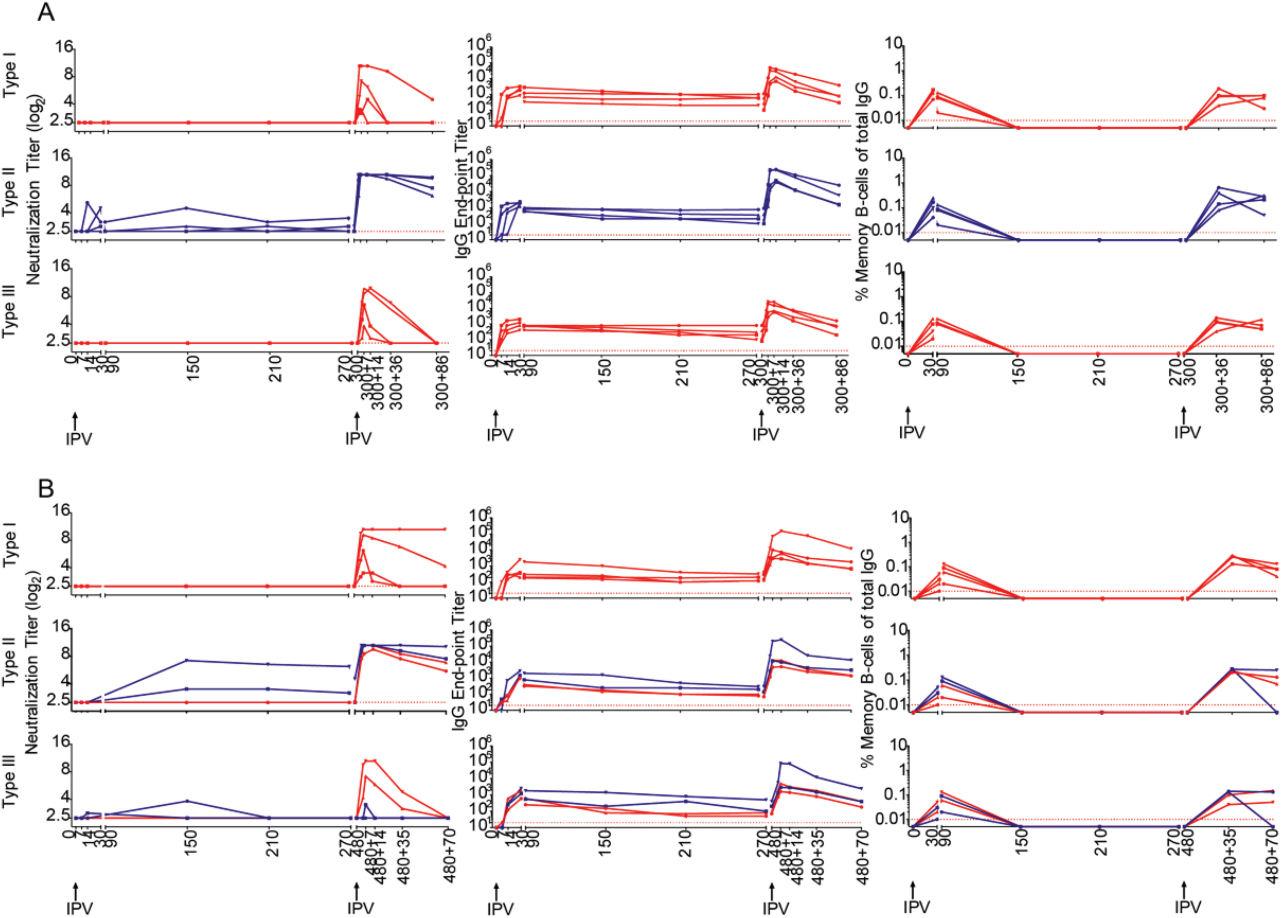
Analysis of long-term persistence of immune priming to each of the poliovirus serotypes long-term post a single full-dose inactivated poliovirus vaccine (IPV) intramuscular ( i.m.). *(A*) IPV challenge was done in 4 macaques 300 days after single full-dose IPV (i.m.). *(B*) IPV challenge was done in 4 macaques 480 days after single full-dose IPV (i.m.). Neutralizing antibody (NT Ab) titers (left panel), immunoglobulin (Ig) G titers (middle panel), and memory B cell frequencies (right panel) are shown for individual macaques. Blue lines indicate those macaques that generated NT Abs after the single IPV immunization. Red lines indicate those macaques that failed to generate neutralization titers after the single IPV immunization. The center panel shows the plasma IgG-binding Ab titers measured in these individual macaques for all 3 poliovirus vaccine antigens; data are represented as the endpoint titer. Abbreviations: Ig, immunoglobulin; IPV, inactivated poliovirus vaccine.

**Figure 8.**
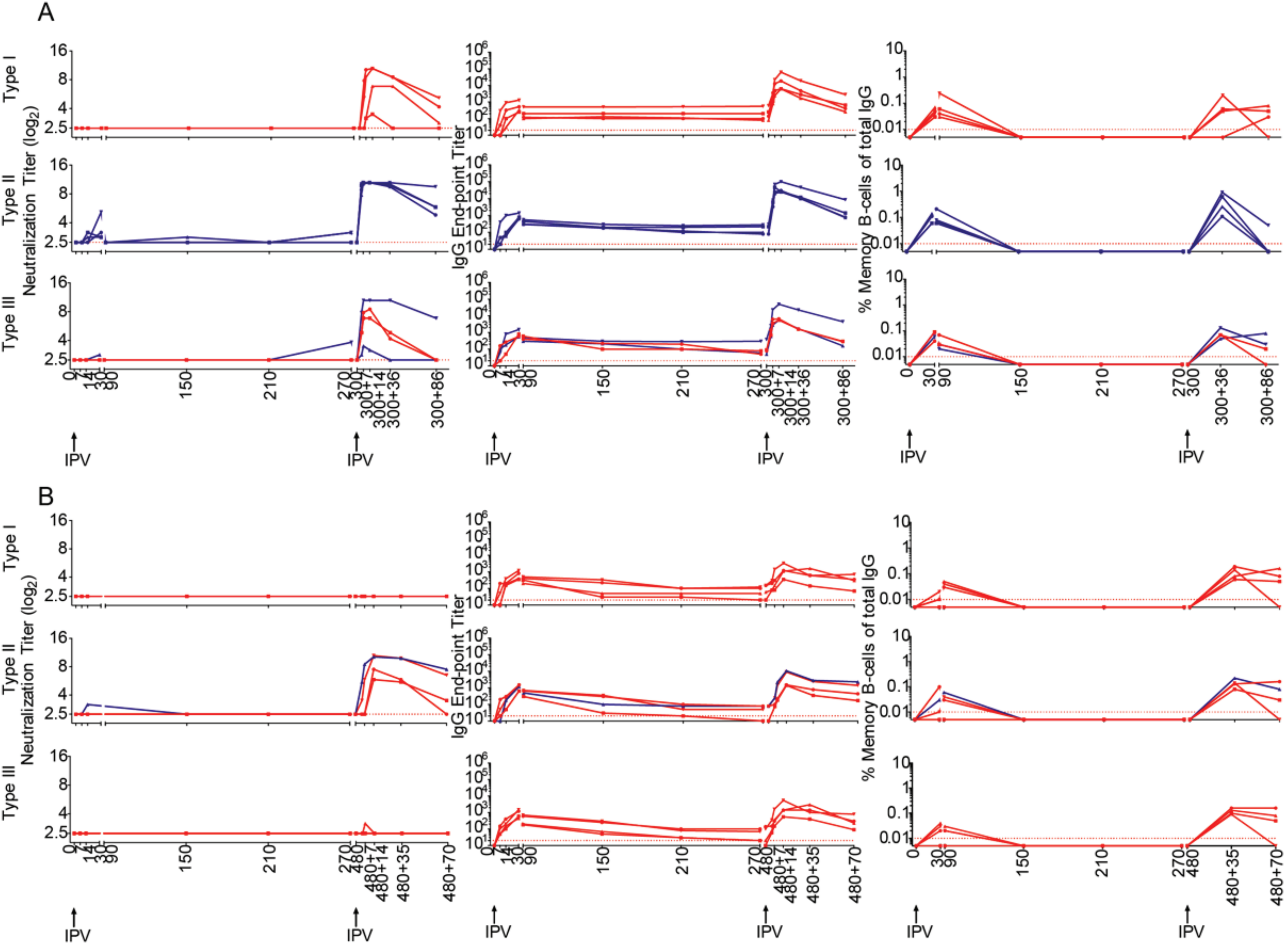
Analysis of long-term persistence of immune priming to each of the poliovirus serotypes long-term post a single fractional-dose inactivated poliovirus vaccine (IPV) intradermal (i.d.). *(A*) IPV challenge was done in 4 macaques 300 days after single fractional-dose IPV (i.d.). *(B*) IPV challenge was done in 4 macaques 480 days after fractional-dose IPV (i.d.). Neutralizing antibody (NT Ab) titers (left panel), immunoglobulin (Ig) G titers (middle panel), and memory B cell frequencies (right panel) are shown for individual macaques. Blue lines indicate those macaques that generated NT Abs after the single IPV immunization. Red lines indicate those macaques that failed to generate neutralization titers after the single IPV immunization. The center panel shows the plasma IgG-binding Ab titers measured in these individual macaques for all 3 poliovirus vaccine antigens; data are represented as the en-point titer. Abbreviations: Ig, immunoglobulin; IPV, inactivated poliovirus vaccine.

To further stringently test whether the immune priming induced by a single dose of IPV completely wanes with time, we rechallenged 4 each of the single full-dose recipient macaques with IPV at days 300 or 480 (Figure 7) and 4 each of the single fractional-dose recipient macaques with IPV at day 300 or 480 (Figure 8). The rechallenge was performed using the same IPV dose and route chosen for the initial priming for each of the groups. At 300 days post single IPV immunization, all the rechallenged macaques elicited NT Abs. This NT Ab response was seen in macaques with measurable NT Abs following the first immunization 300 days previously, as well as in macaques that did not have measurable NT Abs following the first immunization 300 days prior. This NT Ab response was seen against all 3 serotypes and in both single full-dose IPV macaques (Figure 7A) as well as in single fractional-dose IPV animals (Figure 8A). However, the response was more robust against type 2 compared to type 1 and type 3. IgG responses peaked around day 14 in the rechallenged macaques. This is in contrast to the more than 14 days taken for peaking of the IgG response in the first-time IPV vaccine recipients. Taken together, these results suggest that some priming effect persisted for up to 300 days in single-dose IPV recipients, irrespective of whether they had received full- or fractional-dose IPV, and this priming effect was more potent for type 2 than for types 1 and 3. These responses were less efficient in the single-dose IPV macaques that were challenged at day 480 (Figures 7B and 8B). Almost no NT Ab responses were found against type 1 and type 3 viruses at 480 days following single fractional IPV. Taken together, these results suggest that the priming effect induced by a single dose of IPV tends to decline with time and that this time-dependent decline was more evident in the single fractional-dose IPV macaques compared to single full-dose IPV animals.

## DISCUSSION

Sequential removal of OPV types starting with type 2 OPV from routine immunization schedules, as well as the introduction of at least 1 IPV dose from both routine immunization schedules and supplementary immunization activities, was specified in the Polio Endgame Plan [3]. SAGE’s current recommendation is 1 full or 2 fractional IPV doses to provide poliovirus type 2 specific immunity during the period of bOPV usage (which cannot by itself generate type 2 immunity) and to use at least 2 doses of IPV post-OPV1 and 3 withdrawal [22]. Our study is timely and significant in light of these recommendations. It shows the following: 1 dose of IPV fails to induce NT abs in a majority of macaques and, where induced, these NT Abs decline with time; 1 dose of IPV induces memory B cells in all the animals, but these memory B cells fall to below the detection limit in blood circulation by 150 days; a 2-dose schedule can induce NT Abs in all the animals, and these NT Abs persist for at least 420 days in the case of type 2, and for at least 150–420 days in the case of types 1 and 3; the 2-dose schedule induces much higher frequencies of memory B cells compared to the 1-dose schedule, and the memory B cells induced by the 2-dose schedule persist long term in the systemic circulation (for at least 420 days post second dose, the maximum duration tested in these experiments); and full-dose i.m. and fractional-dose i.d. immunization are strikingly similar in these qualities.

However, our study has some limitations. We were able to evaluate only 8 macaques in each group (single full-dose of IPV, single fractional-dose of IPV, 2 full doses of IPV, and 2 fractional doses of IPV), which are small numbers. Larger studies would be useful. Our assays to measure memory B cells are restricted to enumerating the memory B cells that are poised to produce binding antibodies rather than neutralizing antibodies. Our IPV rechallenge experiments suggest that some priming effect is observed with a single dose of IPV, even at 300 and 487 days, although it is inefficient and variable depending upon the serotype specificity, despite our inability to detect blood-circulating memory B cells at these times. Considering these limitations, and considering that the macaque model may not fully mimic human immune responses, careful follow-up studies are warranted in humans to assess long-term maintenance of memory B cells, their ability to produce NT Abs, and their relation to the priming effect, as well as the durability of the priming effect following a single vs 2 doses of IPV.

It is well known that a single dose of IPV does not induce NT Abs in a substantial proportion of human vaccinees [23, 24]. Our macaque data that show that a single dose of IPV does not induce NT abs in the vast majority of the animals are strikingly similar to these observations from humans. Interestingly, we found that a single dose of IPV induces binding antibodies in all animals. This observation raises an interesting question of whether these binding antibodies can be used as a surrogate for assessing whether a protective vaccine-induced immune response has been induced. However, one should exercise caution in extrapolating this result to humans because in humans there is no clear correlation between NT Abs and binding antibodies and humans are more likely to have cross-reactive binding antibodies induced by other enteroviruses circulating in the populations evaluated [13, 24].

Human studies pertaining to long-term persistence of NT Abs were mostly limited to situations where at least 3 or more IPV doses were used. In such conditions, although NT Abs were shown to persist for at least up to 18 years, their titers dropped significantly within the first 2 years post-vaccination [25–30]. To date, human experience with long-term persistence of NT Abs following a 2-dose schedule is limited. Our study highlights 2 important points in this respect: NT Abs are induced consistently in all the vaccinees with a 2-dose schedule and, although there was a substantial drop in titers, these NT Abs were maintained at least for 420 days post second dose against poliovirus type 2 but for much less time (between 150 to 420 days) for types 1 and 3. Corroborating these data, we found that bone marrow ASC seeding was most efficient for poliovirus type 2, followed by types 1 and 3, suggesting that perhaps the bone marrow seeding of ASC is important for long-term maintenance of circulating NT Abs. It is important to compare the length of NT Ab maintenance in humans after these different limited-dose IPV schedules.

Studies suggest that immune priming in the absence of neutralizing antibodies may have limited protective effects against disease [18, 31]; however, to date, no data have been generated related to the durability of this primed immune response in situations where limited doses of IPV were used. In this regard, our findings are important and warrant further studies in humans to assess evolution and long-term maintenance of memory B cells following limited IPV dose schedules.

## Notes

***Acknowledgments.*** The authors thank Emmanuel Vidor (Sanofi Pasteur) for providing purified polio virus antigens. The authors also thank John Modlin and Ananda Bandyopadhyay (Bill & Melinda Gates Foundation) and Stanley Plotkin and Rafi Ahmed (Emory Vaccine Center) for helpful discussions and encouragement.

***Financial support.*** This work was supported by the World Health Organization Global Polio Eradication Initiative, the Monell Foundation, and the Emory Vaccine Center, Emory University School of Medicine.

***Supplement sponsorship.*** This article appears as part of the supplement “Polio endgame and beyond: Vaccine choices, transmission dynamics, and surveillance implications,” sponsored by The Bill & Melinda Gates Foundation.

***Potential conflicts of interest.*** All authors: No reported conflicts of interest. All authors have submitted the ICMJE Form for Disclosure of Potential Conflicts of Interest. Conflicts that the editors consider relevant to the content of the manuscript have been disclosed.
